# Adrenal Dysfunction in Mitochondrial Diseases

**DOI:** 10.3390/ijms24021126

**Published:** 2023-01-06

**Authors:** Madeleine Corkery-Hayward, Louise A. Metherell

**Affiliations:** 1 Barts and the London Medical School, Queen Mary University of London, London E1 2AD, UK; 2 Centre for Endocrinology, William Harvey Research Institute, Queen Mary University of London, London EC1M 6BQ, UK

**Keywords:** adrenal insufficiency, mitochondria, familial glucocorticoid deficiency, mitochondrial diseases

## Abstract

Cortisol is central to several homeostatic mechanisms including the stress and immune response. Adrenal insufficiency and impaired cortisol production leads to severe, potentially fatal disorders. Several fundamental stages of steroidogenesis occur within the mitochondria. These dynamic organelles not only contribute ATP for steroidogenesis, but also detoxify harmful by-products generated during cortisol synthesis (reactive oxygen species). Mutations in nuclear or mitochondrial DNA that impair mitochondrial function lead to debilitating multi-system diseases. Recently, genetic variants that impair mitochondrial function have been identified in people with isolated cortisol insufficiency. This review aimed to clarify the association between mitochondrial diseases and adrenal insufficiency to produce cortisol. Mitochondrial diseases are rare and mitochondrial diseases that feature adrenal insufficiency are even rarer. We identified only 14 cases of adrenal insufficiency in people with confirmed mitochondrial diseases globally. In line with previous reviews, adrenal dysfunction was most prevalent in mitochondrial deletion syndromes (particularly Pearson syndrome and Kearns–Sayre syndrome) and with point mutations that compromised oxidative phosphorylation. Although adrenal insufficiency has been reported with mitochondrial diseases, the incidence reflects that expected in the general population. Thus, it is unlikely that mitochondrial mutations alone are responsible for an insufficiency to produce cortisol. More research is needed into the pathogenesis of adrenal disease in these individuals.

## 1. Introduction

Steroids are vital hormones implicated in a plethora of homeostatic pathways that are necessary for normal physiological function [1]. Mitochondria are the main source of cellular ATP, and are therefore integral cellular components. They are also crucial for steroidogenesis, as in addition to providing ATP, they are also the site of key rate limiting steroidogenic reactions [2]. Both dysfunctional mitochondria and deranged steroidogenesis can precipitate fatal multi-system disorders. However, the exact role of mitochondria, particularly mitochondrial dysfunction, in the development of sterol insufficiency remains unclear [3,4].

## 2. Adrenals

The adrenals are two small encapsulated endocrine glands located above the kidneys [5]. They have a central medulla, composed of chromaffin cells, which is surrounded by three adjacent layers (the cortex) that produce steroids from cholesterol [5]. These layers are protected by an outer adrenal capsule that also contributes to adrenal cell renewal [5,6]. Each adrenal layer acts as a morphologically distinct functional zone [6]. The outermost zone, the zona glomerulosa (zG), produces mineralocorticoids responsible for regulating blood pressure, salt and water balance (through the renin–angiotensin–aldosterone system) [6,7]. The adjacent zona fasciculata (zF) produces glucocorticoids (GC). These regulate the immune and stress response [5,6]. The zona reticularis (zR) produces androgens and surrounds the adrenal medulla, which lies at the centre of the adrenal gland and produces noradrenalin/adrenalin [5,7]. Interestingly, mouse models indicate that both zF and zG cells arise from the same foetal precursor cells. Consequently, their steroidogenic pathways are very similar [7].

### 2.1. Cortisol

The zona fasciculata (zF) comprises 80% of the cortex—reflecting the higher physiological demand for GC compared to the other adrenal steroid hormones [7]. Cortisol is the most abundant GC and its diverse physiological effects are mediated through glucocorticoid receptors that are ubiquitously expressed (GR) [8,9]. Sufficient foetal cortisol production is crucial for normal organ system development [5]. Postnatally, it is involved in multiple homeostatic mechanisms including regulating metabolism and immune responses [6]. Cortisol rapidly mobilises glucose stores and increases cardiac output, enabling the sympathetic stress response [9,10]. Recent research also suggests that it plays an additional immunomodulatory role and directs T-cell response and migration during acute illness [9]. 

### 2.2. HPA Axis Regulation of Cortisol

Due to its vital regulatory role, cortisol synthesis is tightly regulated. Hormonally, it is controlled by the hypothalamic–pituitary–adrenal (HPA) axis [6,11]. Stress or low circulating cortisol prompts the hypothalamus to secrete corticotropin releasing hormone (CRH), which acts on the anterior pituitary, to release adrenocorticotropic hormone (ACTH) into the bloodstream [11]. Within the adrenals ACTH binds to melanocortin-2-receptor (encoded by *MC2R*), initiating the complex intracellular cascade for cortisol biosynthesis [8,11,12]. Most MC2R are located in the zF and zG, and thus their activation is crucial for aldosterone and cortisol production [11,13]. MC2R are synthesised in the ER and can only be transported to the cell membrane by the transmembrane protein melanocortin-2 receptor accessory protein (*MRAP*) [11]. Non-sense mutations that truncate *MRAP* result in ACTH insensitivity and severe early onset complete glucocorticoid deficiency [8,11,14]. Similarly *MC2R* mutations have also been implicated in the development of isolated GC deficiency (known as ‘familial glucocorticoid deficiency’, FGD—see Appendix A) [15].

## 3. Steroidogenesis—Cholesterol Import and Mobilisation

As with all steroid hormones, cortisol is generated from cholesterol (a process dubbed ‘steroidogenesis’) [8]. Consequently, intracellular cholesterol concentrations are tightly regulated and linked to sterol demand [8]. In times of low dietary intake but robust sterol demand, dietary cholesterol is endocytosed with HDLs and LDLs and released by hormone sensitive lipase (HSL) or lysosomal acid lipase (LAL), respectively [2,8]. Low circulating cortisol also stimulates ACTH release, which in turn increases adrenal cell LDL endocytosis, thereby increasing cholesterol bioavailability for steroidogenesis—the first stage of which takes place within the mitochondria [2,8].

## 4. Steroidogenesis and Mitochondria

### 4.1. Mitochondrial Membranes 

Mitochondria are vital for steroidogenesis as the location of several key biosynthetic stages, with the rate limiting step in cortisol production occurring across the mitochondrial membranes [16]. These consist of a barrier of an outer mitochondrial membrane (OMM) and an inner mitochondrial membrane (IMM), which invaginates to form cristae [17]. Mitochondrial membranes are dynamic, regularly fusing and reforming with other organelles and mitochondria. This flexibility is essential for regulating multiple key signalling and homeostatic pathways [17].

**Figure 1:**
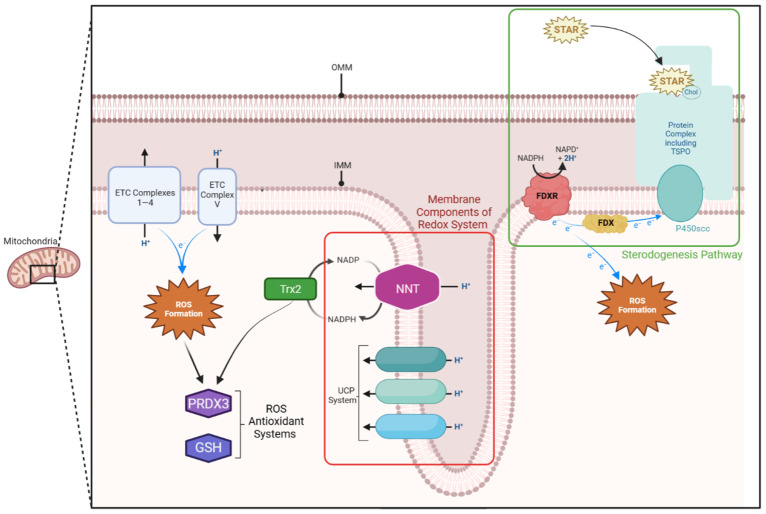
Mitochondrial membrane components of the steroidogenic and redox pathway. The translocator protein (TSPO) complex lies on the outer mitochondrial membrane (OMM) and allows cholesterol to enter the intermembranous space. Here, the steroidogenic acute regulatory protein (STAR) shuffles it across to the cholesterol side-chain cleavage enzyme (P450scc) on the inner mitochondrial membrane (IMM). P450scc relies on a continuous supply of electrons, which are provided by the ferredoxin reductase (FDXR)—ferredoxin (FDX) system on the IMM. Both the electron transport chain (ETC) and FDXR-FDX system produce reactive oxygen species (ROS). These are detoxified by thioredoxin-dependent peroxide reductase (PRDX3), reduced glutathione (GSH), and thioredoxin-2 (Trx2) systems within the mitochondria, which rely on nicotinamide adenine dinucleotide phosphate (NADPH). The FDXR-FDX system is similarly reliant on NADPH. This NADPH supply is replenished by the nicotinamide nucleotide transhydrogenase (NNT) protein, located on the inner mitochondrial membrane (IMM). The uncoupling protein (UPC) also acts as an antioxidant by diverting protons from the intermembranous space back into the matrix. This diverts them from the terminal protein in the electron transport chain, thereby reducing ROS production.

Zona fasciculata cells do not store large amounts of cortisol and sudden increases in steroid demand such as in response to stress therefore require rapid cortisol synthesis and cholesterol delivery to P450scc on the IMM [2,8]. On the IMM, the cholesterol side-chain cleavage enzyme, cytochrome P450scc, cleaves cholesterol to pregnenolone (the rate limiting step in steroidogenesis) (see Figure 1) [2,16]. To facilitate this reaction, the steroidogenic acute regulatory D4 (STARD4) binding protein shuttles cholesterol through the cytosol to mitochondria, where the steroidogenic acute regulatory proteins (STAR) move it across the mitochondrial membranes [2,8,18].

### 4.2. Cholesterol Shuttle

STAR is therefore essential for maintaining steroidogenesis in adrenal cells, with rare mutations that decrease or abolish STAR bioactivity highlighting this. The consequential decrease in steroid hormones increases intracellular lipids, eventually resulting in mitochondrial and cell death [2,3,8]. The resultant disease, known as congenital lipoid adrenal hyperplasia (lipoid CAH), is potentially fatal, presenting with critical adrenal failure in the first few months of life [18,19]. STAR is a 37-kDa protein, and is cleaved to its ‘mature’ form within the mitochondria before localising to the OMM [2,8,20]. Surprisingly, it is not cleavage to a ‘mature’ form that increases STAR activity, but the time in contact with the OMM [2]. The OMM’s acidic microenvironment prompts an additional conformational change, exposing STAR’s cholesterol binding site [2]. Thus, mitochondrial membranes regulate the rate-limiting step in steroidogenesis as it is the OMM that ultimately ensures STAR activity.

It has long been thought that STAR acted alone to shuttle cholesterol across the mitochondrial membranes. However, recent in vivo studies have also implicated a complex including the translocator protein (TSPO) [2,21,22]. TSPO constitutes roughly 2% of the OMM and complexes with several other mitochondrial membrane proteins (including STAR). It is this complex that facilitates cholesterol movement to P450scc on the IMM [2,21,23]. As with STAR, TSPO activity is dependent on the OMM composition. This highlights the importance of the mitochondrial membrane composition for cortisol synthesis.

### 4.3. The Start and the End

P450scc converts cholesterol to pregnenolone in a three stage reaction on the IMM [2]. P450scc is the only enzyme able to catalyse this reaction and is coded for by the *CYP11A1* gene, on chromosome 15q24.1 [2]. As this conversion to pregnenolone is necessary for all steroid production, a cell is only considered steroidogenic if it expresses the *CYP11A1* gene (and consequently P450scc) [8]. Interestingly, P450scc can only function within the mitochondria, further cementing them as essential for steroidogenesis. Once synthesised, pregnenolone diffuses out of the mitochondria unaided and enters the ER. Here, a series of biosynthetic reactions produce 11-deoxycortisol in the zF and deoxycorticosterone in the zG [2,8]. They then re-enter the mitochondria, where the enzyme steroid 11β-hydroxylase (P450c11β), encoded by *CYP11B1* and *CYP11B2*, catalyses the final step in steroidogenesis—conversion to cortisol and aldosterone, respectively [2]. Interestingly, the imaging of zF cells showed that the ER briefly complexes with the OMM, and it is this that enables 11-deoxycortisol and deoxycorticosterone to return to the mitochondria [2,8]. Again, this demonstrates the importance of the mitochondrial membranes for cortisol production.

### 4.4. Genetic Profiling

The pivotal role of each of these proteins in steroidogenesis is evident from the genetic investigation of individuals with FGD. Candidate gene studies in 1993 first identified a point mutation in the *MC2R* gene [24]. Subsequent screening soon implicated a second gene, *MRAP* [25]. *MC2R* is the most common genetic mutation in FGD—over 40 different *MC2R* mutations have been reported (accounting for 25% of FGD cases, termed FGD Type 1) [15]. Most are non-conservative missense mutations, which alter the *MC2R* protein structure so that it remains sequestered within the endoplasmic reticulum with a minority affecting ligand binding [8,12,15].

*MRAP* variants present earlier than *MC2R* mutations (often within one month of birth). People with *MRAP* mutations (termed FGD Type 2) account for 20% of solved FGD cases and typically have a more severe phenotype [12,14,15]. These splice site or non-sense mutations produce truncated *MRAP* proteins, which lack key transmembrane domains and are non-functional [14,15]. This non-functional *MRAP* cannot accompany *MC2R* out of the ER, decreasing membrane *MC2R* [15]. Both *MC2R* and *MRAP* mutations ultimately reduce ACTH signal transduction across the zF cell membrane [3]. Although *MC2R* and *MRAP* are expressed at low levels throughout the adrenals, they are particularly abundant in the zF [11,15,26]. Given this, it is unsurprising that the GC pathway is particularly affected by *MRAP* and *MC2R* mutations, causing isolated GC deficiency.

STAR mutations are classically associated with syndromic lipoid CAH and disordered sexual development [27]. However, there are a number of reports of partial loss-of-function STAR variants that also cause non-classical FGD (with preserved mineralocorticoid and androgen production) [15]. Mutations in *CYP11A1* also typically produce a syndromic picture similar to lipoid CAH, with adrenal and gonadal steroid insufficiency and atypical sexual development [3,8,11]. However, multiple loss of function *CYP11A1* mutations are also implicated in the development of isolated GC deficiency [11,15,28,29]. Both STAR and *CYP11A1* are particularly abundantly expressed in the adrenal cortex [7,15]. Given their central role in steroid biosynthesis and the higher demand for cortisol over other adrenal steroid hormones, it is unsurprising that *CYP11A1* and STAR defects can present with isolated cortisol derangement (FGD) [15]. It is now estimated that *CYP11A1* and STAR variants are the underlying cause of up to 10% of FGD cases [11].

### 4.5. P450 Enzymes

Steroidogenic enzymes can be broadly divided into two classes (hydroxysteroid dehydrogenases and cytochrome P450s), which are spread between the ER and mitochondria. The mitochondria contain six P450 enzymes (Type 1), whose function depends on a series of reduction–oxidation (redox) reactions facilitated by ferredoxin reductase (FDXR) and ferredoxin (FDX) [2,8,20]. Both of these proteins lie on the IMM [20,23]. Here, they shuttle electrons from NADPH in a chain to P450scc [20,23]. Although the FDXR-FDX chain is ideally a closed system, electrons often ‘escape’, producing harmful reactive oxygen species (ROS) [2,8,20,23]. Reflecting the extreme electron demand of P450 enzymes, concentrations of FDXR are a hundred times higher in steroidogenic cells [2]. Consequently, steroidogenesis produces large amounts of ROS, mainly concentrated in the mitochondria.

### 4.6. Mitochondrial Reactive Oxygen Species

ROS are unstable oxygen derivatives generated through aerobic metabolism [23,30]. Extensive research indicates that they have paradoxical physiological effects [30]. ROS are key signalling molecules for many vital cellular pathways, however, in excess, ROS also appear to cause widespread damage (termed ‘oxidative stress’) and cell death [23,30]. Electron loss is not uniform in P450 enzymes; the reduction chain for P450scc is relatively conservative, losing only 15% of its electrons [23]. In contrast, 40% of the electrons associated with the terminal *CYP11B1* (the last enzyme in cortisol synthesis) enzyme ‘escape’ [23]. Consequently, pathways involving P450c11β may increase the levels of ROS above that of other steroidogenic pathways, subjecting zF cells to increased oxidative stress. Due to their destructive potential, ROS are tightly regulated (‘redox regulation’). Impaired ROS regulation is implicated in the pathogenesis of multiple diseases including FGD [23,30]. Workup of genetically unmapped people with FGD identified novel mutations in two mitochondrial genes (*NNT* and *TXNRD2*—see below), and these may have impaired redox regulation [15,16].

## 5. Mitochondria and Oxidative Stress

### 5.1. Antioxidant Mechanisms

Mitochondria are central to cellular redox regulation. As the site of aerobic respiration, they are leading contributors to ROS production, primarily hydrogen peroxide (H_2_O_2_) [16,17,23,30,31]. Most cellular ROS are generated through unintentional electron ‘escape’ at respiratory chain complexes I and III on the IMM [16,23,30]. However, adrenal tissues face an additional ROS burden from p450 enzymes (particularly P450c11β [23] and have a number of antioxidant systems to compensate for this, in particular by specific peroxidases at the expense of reduced glutathione and thioredoxin.

### 5.2. Reduction Systems

Reduced glutathione (GSH) and thioredoxin-2 (Trx2) are two key mitochondrial antioxidants. They localise to the IMM and their regulation of ROS is vital for normal cell function and steroidogenesis [23,23,31,32]. GSH and thioredoxin-2 are reduced by NADPH. This NADPH supply is largely replenished by nicotinamide nucleotide transhydrogenase (NNT) [23,30].

Ample NADPH is also essential for the P450 linked FDXR–FDX complex. Given the high P450 activity and ROS production in steroidogenesis, it is unsurprising that NADPH concentrations are between 10 and 100 times higher within the mitochondria than in the cytosol [32]. As a key NADPH supplier, zF is therefore heavily reliant on NNT. Murine modelling corroborates this, as does knockdown of NNT in H295R human adrenal cells. Loss of NNT and the subsequent decrease in GSH demonstrated that low levels of mitochondrial GSH increased ROS formation, which then directly decreased steroidogenesis [23,30,33].

**Figure 2:**
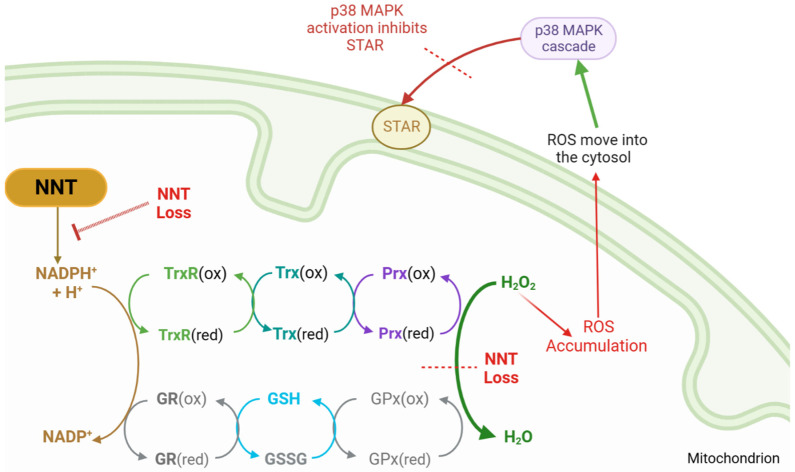
PRDX3 steroidogenesis feedback loop, outlining reduction–oxidation reactions that detoxify reactive oxygen species (ROS). Nicotinamide nucleotide transhydrogenase (NNT) supplies the reductive power for the glutathione (GSH/GSSG) and thioredoxin (Trx) systems to detoxify hydrogen peroxide (H_2_O_2_). With NNT loss, ROS accumulates and leaks into the cytosol, where it activates the p38 MAPK cascade, inhibiting STAR function and reducing cholesterol transfer. NNT = nicotinamide nucleotide transhydrogenase, NADP = nicotinamide adenine dinucleotide phosphate, NADPH = reduced NADP, MAPK = mitogen-activated protein kinase, STAR = steroidogenic acute regulatory protein, GSH = reduced glutathione, GSSG = glutathione disulphide, GR = glutathione reductase, GPx = glutathione peroxidase, Trx = thioredoxin, TrxR = thioredoxin reductase, Prx = peroxiredoxin, (ox) = oxidated state, (red) = reduced state.

Thioredoxin reductase 2 (TrxR2) interacts with thioredoxin-dependent peroxide reductase ‘scavenger’ proteins (PRDX3 and PRDX5) located in the mitochondrial matrix [23]. PRDX3 is the primary mitochondrial antioxidant, detoxifying around 90% of the mitochondrial H_2_O_2_ into H_2_O [34]. Interestingly, animal modelling of the TrxR2 system indicates an inverse relationship between redox regulation and steroidogenesis [35]. In murine adrenal cells, P450c11β appears to directly inactivate PRDX3, converting it to a hyperoxidized state [23]. High rates of cortisol synthesis increase PRDX3 inactivation. Consequently, H_2_O_2_ accumulates in the mitochondria and diffuses into the cytosol [35]. There, excessive H_2_O_2_ triggers the p38 MAPK signalling cascade, directly inactivating STAR [16,23]. Other steroidogenic proteins are also particularly susceptible to this increase in oxidative stress, further impacting steroidogenesis [23]. It is not yet clear whether this decrease results from direct protein damage or the effects on transcription [23]. However, through these two mechanisms, PRDX3 forms part of a mitochondrial specific self-regulatory cortisol feedback loop (Figure 2).

### 5.3. IMM Proton Carriers

The mitochondrial ‘UCP system’ has also been implicated in ROS clearance [31]. This consists of three IMM proteins linked to the aerobic electron transport chain (ETC) [31]. Uncoupling protein 1 (UCP1 or ‘thermogenin’) diverts protons from the intermembranous space to the matrix, so they bypass the terminal ETC complex (V), thereby decreasing the IMM potential [31]. This decreases the ETC activity, with a consequential reduction in ROS formation. UCP2 and -3 similarly lower ROS production, although the precise ROS sequestering mechanisms are still debated [31]. Given the additional ROS burden generated in the adrenals by P450 enzymes, the IMM UCP system may be particularly important for adrenal redox regulation and normal cortisol production.

## 6. Genetic Profiling of Mitochondrial Genes

Surprisingly, alongside those classically associated with FGD, ubiquitously expressed genes linked to mitochondrial redox regulation are linked to isolated cortisol deficiency [15].

### 6.1. NNT

As previously outlined, the NNT protein is essential for NADPH repletion within the mitochondria and so is highly conserved across all human tissues [15,36]. However, NNT mutations are now thought to account for 5–10% of FGD cases [36]. A review in 2015 described 27 frameshift and missense NNT mutations in a cohort of people with FGD [36,37]. The resultant protein truncations led to either non-functional proteins or the loss of integral signalling domains that prevented NNT entering the mitochondria [36,38].

Evidence from animal models demonstrates that the loss of NNT decreased mitochondrial concentrations of GSH, thereby increasing cell susceptibility to oxidative stress [38]. In vitro NNT knockdown in human adrenocortical H295R cells also produced a global NADPH deficit, leading to lower GSH and higher ROS levels with a consequential increase in mitochondrial ROS and apoptosis [15,38]. This reaffirms the essential role of mitochondrial NNT and GSH in redox regulation and provides some insight into how mutated NNT may cause FGD [11].

There is a higher physiological demand for cortisol than other adrenal steroid hormones, and consequently, harmful ROS may accumulate faster in zF cells, subjecting them to increased oxidative stress. Non-NNT linked antioxidant mechanisms may be unable to detoxify all the ROS produced by *CYP11B1* during cortisol biosynthesis without the NNT system. This would explain the initial and clinical picture seen with most NNT mutations; deranged cortisol levels with preserved mineralocorticoid and adrenal androgen production (FGD) [11].

### 6.2. TXNRD2

Discovery of these NNT mutations has prompted investigation into other redox related genes [15,39]. This identified a novel homozygous stop gain mutation p.Y447X in *TXNRD2*, causing nonsense mediated decay and TrxR2 depletion [39]. As previously mentioned, TrXR2 works alongside NNT in adrenal cell antioxidant pathways [23,39]. Although *TXNRD2* is conserved across all human tissues, expression profiling shows that it is most concentrated in human adrenal cells and *TXNRD2* knockout precipitates adrenal failure [23]. This disproportionate impact on human adrenal cells may be due to their particular dependence on redox regulation [39].

### 6.3. Syndromic Presentations

It is well-established that increased oxidative stress leads to many other syndromic diseases with adrenal insufficiency [3,11,40].

Triple A syndrome is one such disease precipitated by autosomal recessive mutations in the *AAAS* gene. It presents with ACTH resistance with the triad of achalasia, alacrimia, and GC deficiency, which is a key FGD differential [8,11,23,41]. *AAAS* encodes ALADIN, a nucleoporin required for redox regulation [8]. Defects in this gene augment cellular oxidative stress, producing diverse pleiotropic effects including adrenal insufficiency [8,39].

Recently mutations in sphingosine-1-phosphate lyase (SGPL1) have been linked to adrenal insufficiency with treatment resistant nephrotic syndrome, dubbed sphingosine phosphate lyase insufficiency syndrome (SPLIS) [11,42]. SGPL1 is heavily involved in steroidogenesis, but is also implicated in several other cellular processes including redox regulation [11]. SGPL1 catalyses the terminal irreversible step in the sphingolipid degradation pathway, thereby controlling the cellular concentrations of harmful sphingolipid intermediates (such as ceramide) [43]. Ceramide acts as a key second messenger for steroidogenesis, decreasing steroid hormone production. Increases in SGPL1 activity reduce cellular levels of ceramide, and consequently increases cortisol biosynthesis [43]. Pathological accumulation of sphingolipid intermediates disrupts the IMM, causing mitochondrial dysfunction [43]. As established, the IMM is the location of many key mitochondrial redox systems including the UCP and NNT proteins. It is therefore possible that this IMM damage incidentally impairs mitochondrial ROS handling, increasing oxidative stress. SGPL1 mutations result in wide ranging, diverse phenotypes with severe multisystem pathologies including deranged neurodevelopment, hypothyroidism, and dyslipidaemia [11]. However, SPLIS is the only sphingolipidoses that has presented with multi-endocrine dysfunction (primary adrenal insufficiency (PAI) and hypothyroidism) [42,44].

## 7. Literature Review

As established, mitochondria are essential for redox regulation in all human tissues and, as the site of the initial and terminal reactions in cortisol synthesis, they are crucial for steroidogenesis [40]. The phenotypes of mutant NNT and *TXNRD2* demonstrate that perturbations in redox homeostasis within mitochondria can affect steroidogenesis. As these variations disrupt mitochondria metabolism, the resultant syndromes can be considered mitochondrial metabolic disorders [3].

### Syndromes and Endocrine Abnormalities

Mitochondrial disorders are a heterogenous group of diseases characterised by mitochondrial ETC and/or metabolic dysfunction due to mutated nuclear or mitochondrial DNA (mtDNA) [40].

This expansive category includes clinical syndromes such as MELAS (mitochondrial encephalopathy, lactic acidosis and stroke-like episodes) as well as diseases defined by pathogenic loss of mtDNA including Pearson syndrome (PS) and Kearns–Sayre syndrome (KSS) [3]. Mitochondropathies are often syndromic, and adrenal insufficiency is rare (despite the reliance on mitochondria for steroidogenesis) [40,45,46]. Thus, it is surprising that people with mutations in NNT and *TXNRD2* present with adrenal, but no other system, dysfunction. These unexpected findings prompted a literature review to explore the association between mitochondrial disease and primary adrenal insufficiency (PAI).

## 8. Literature Review Results

Adrenal insufficiency is not commonly associated with mitochondrial diseases. Of the roughly 1:5000 people affected by mitochondrial diseases globally, there are currently only 23 reports of people with concurrent adrenal dysfunction, as summarised in Table 1 [40].

This search updates a review by Calderwood et al. in 2015, which identified 12 case reports of mitochondrial diseases with concurrent PAI (14 people) [4]. Notably, PAI is rare, and was not the initial presentation in any case and followed systemic multi-system dysfunction. Indeed, of the endocrine disorders linked to mitochondrial diseases, diabetes mellitus was the most common, and PAI the least [4]. The age of onset of adrenal insufficiency varied widely, ranging from 7 months to 32 years, occurring alongside a constellation of other clinical features (Table 1) [4].

Eight cases were autoimmune in origin (in three of these PAI was part of an autoimmune polyendocrine syndrome) [47,48,49]. One other report described ACTH deficiency, not PAI, and in three cases, the cause of adrenal insufficiency was unknown [50,51,52].

### 8.1. Patient Registries

The North American Mitochondrial Disease Consortium (NAMDC) Patient Registry records all cases of mitochondrial disease in North America (n = 634) [45]. MELAS, PS, and KSS were the mitochondrial disorders that most frequently presented with endocrine abnormalities, but only three of cases featured adrenal insufficiency (MELAS = 2, PS/KSS = 1) [45].

A 2017 review of this registry noted that adrenal insufficiency was deemed so uncommon as to be excluded from the pre-specified list of linked endocrine complications [45]. As ‘adrenal insufficiency’ therefore had to be entered as additional information, it is impossible to determine the exact nature of adrenal dysfunction in these three cases [45]. A cohort study across 55 centres in Italy also linked PS with adrenal insufficiency [52]; of the eleven patients reported to have PS, only two suffered from complete adrenal insufficiency, although again, they did not describe the exact nature of adrenal dysfunction [52].

### 8.2. mtDNA Deletion Syndromes

Large-scale mtDNA deletion syndromes are the most common mitochondropathies associated with adrenal insufficiency [40]. Of the 23 reports of adrenal dysfunction in mitochondrial disease, 11 resulted from mtDNA deletions (Table 1), most often PS and Kearns–Sayre syndrome (five and two patients, respectively [4,49,50,53,54,55,56,57,58,59].

Interestingly, deletion size and presentation severity or age of onset of PAI do not appear to correlate. mtDNA deletions linked to PAI ranges from 1.5 kb to as large as ~9 kb, and the age of onset is similarly varied [58,60]. A Korean neonate with PS developed adrenal insufficiency aged six and genetic analysis revealed a mtDNA deletion of 2.3 kb [51]. However, a much larger 9 kb deletion was detected in a male with KSS, who presented aged five with a similar phenotype of PAI on a background of multiorgan system failure [58].

Currently, there is only one reported case of someone with adrenal insufficiency as the presenting complaint [54]. In 1997, Nicolino et al. described a four year old female with complete adrenal failure [54]. Although PAI dominated her phenotype in childhood, she later developed multiorgan failure [54]. Subsequent genetic investigation revealed a 7.4 kb mtDNA deletion in mtDNA [54]. This ~7 kb deletion is common in mitochondrial deletion syndromes, typically presenting with rapid neuromuscular decline [54]. Although she later developed myopathy and ataxia, the initial presentation of adrenocortical insufficiency remains unique in the literature and was attributed to a potentially higher accumulation of mutant mtDNA in the adrenal cortex compared to neuromuscular tissues [54].

Interestingly, this disease progression is remarkably like that of individuals known to have SGPL1 deficiency [43,54]. Although mtDNA was extensively investigated, she did not have whole exome sequencing to investigate other known monogenetic causes of PAI. Therefore, an undetected SGPL1 mutation, or another known variant, may underlie her adrenal insufficiency.

### 8.3. Mutations in Nuclear DNA

Alongside deletion syndromes, mitochondrial disorders due to nuclear DNA mutations are also linked to adrenal insufficiency (see Table 1), although more tenuously [40].

A proposed pathogenic *POLG1* p.Gly517Val mutation was identified in identical twins with diabetes mellitus, neuronal disturbance, and PAI [48]. Endocrine involvement (particularly diabetes mellitus) is known to occur in people with POLG mutations [48]. However, these are currently the only reported cases of a POLG mutation with PAI [61]. The role of this mutation in the development of PAI is uncertain as the same mutation was also present in four other patients who did not develop adrenal insufficiency [62]. Furthermore, analysis of the mutant *POLG1* enzyme indicated that it only lost 10–20% of its functionality, and thus was likely a benign mutation [40,62].

Nine reports link adrenal insufficiency with point mutations that impair ETC complex activity (Table 1) [4,63,64,65]. The age of adrenal insufficiency varied (7 days to 16 years) and it was always part of a syndromic picture dominated by other organ-system derangement [64]. Of note, only four cases had confirmed PAI [53,63,66]. The underlying pathogenic mechanism remained elusive, with authors acknowledging that undiscovered mutations may be responsible for the adrenal malfunction [53,63,66].

There were two reports of adrenal insufficiency due to defective mtDNA translation [67,68]. A 16 year old female with defects in the *MRPS7* gene (c.550A>G, p.Met184Val) developed adrenal insufficiency alongside sensorineural deafness, lactic acidosis, and primary hypogonadism [67]. However, her sibling had the same mutation and a more severe phenotype (with hepatic and renal failure), but normal adrenal function [67]. This casts doubt on the role of this mutation in the development of her adrenal insufficiency.

A homozygous *IARS2* missense mutation likewise resulted in an adrenal insufficiency in a 20.6 year old male [68]. Again, the link between this variant and adrenal insufficiency is questionable. *IARS2* encodes mitochondrial tRNA and mutant *IARS2* inhibits the synthesis of crucial mitochondrial proteins. A review of *IARS2* mutations highlighted that this was the only case with adrenal insufficiency [68]. As the proband also had growth hormone deficiency, the authors could not rule out excluding the possibility of secondary adrenal failure due to HPA axis dysfunction [68]. 

## 9. Discussion

**Figure 3:**
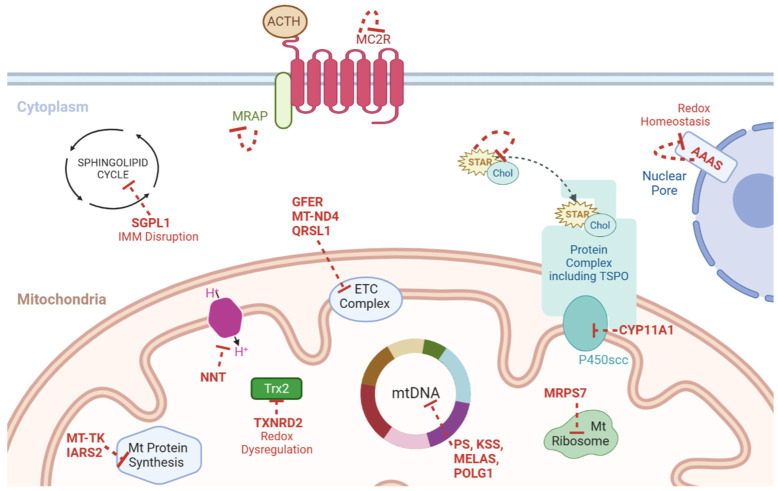
Schematic representation of the genes linked to PAI and mitochondrial dysfunction (genes and the effect of their loss denoted in red). ACTH = adrenocorticotrophin, Chol = cholesterol, ETC = electron transport chain, KSS = Kearns–Sayre syndrome, Mt = mitochondria, mtDNA = mitochondrial DNA, NNT = nicotinamide nucleotide transhydrogenase, PS = Pearson syndrome, TSPO = translocator protein, MRAP = Melanocortin-2 receptor accessory protein, MC2R = Melanocortin 2 Receptor gene, SGPL1 = Sphingosine-1-Phosphate Lyase 1, IMM = Inner Mitochondrial Membrane, GFER = Growth factor, augmenter of liver regeneration, MT-ND4 = Mitochondrially encoded NADH dehydrogenase 4, QRSL1 = Glutaminyl-TRNA Aminotransferase Subunit QRSL1, MELAS = Mitochondrial Encephalopathy, Lactic Acidosis, and Stroke-like episodes, POLG1 = DNA polymerase subunit gamma, TXNRD2 = Thioredoxin Reductase 2, MT-TK = Mitochondrially encoded tRNA lysine, IARS2 = Isoleucyl-TRNA Synthetase 2, MRPS7 = Mitochondrial Ribosomal Protein S7, CYP11A1 = Cytochrome P450 Family 11 Subfamily A Member 1, STAR = Steroidogenic Acute Regulatory Protein, AAAS = Gene encoding Aladin protein.

Mutations that manifest with PAI affect multiple pathways (see Figure 3) and organelles including mitochondria, with most due to monogenic gene mutations. However, a small number of cases may be due to large scale mtDNA rearrangements or deletions.

### 9.1. Deletion Syndromes

In line with previous reviews, mitochondrial deletion syndromes (particularly, PS and KSS) were most frequently linked to adrenal insufficiency [4,53]. The phenotypes of mitochondrial deletion syndromes fall along a spectrum, with large overlap [55]. Pearson syndrome (PS) is a rare multi-organ mitochondropathy (with <100 cases globally) [51]. The most common deletion size was 4.98 kb (45% of cases) [52,59]. Interestingly, no-one with adrenal dysfunction had a deletion of this size and none reported which genes were affected in these patients [51,59]. Two reports did not specify the size of the deletion, and the remaining two cases had deletions of 5.1 kb and 2.3 kb [51,52,59].

The stereotypical presentation of PS is based on this ‘typical’ 4.98 kb deletion (see Appendix B). Given that the patients with adrenal insufficiency had ‘atypical’ deletions, it is unsurprising that they also had atypical presentations (namely adrenal failure). However, all cases of PS with adrenal failure presented with a combination of classical symptoms, which preceded adrenal dysfunction. In total, only four cases of PS had adrenal insufficiency, corroborating previous findings that indicate that in PS, endocrine involvement outside the pancreas is uncommon [52].

KSS is also a progressive mitochondropathy that results from mtDNA deletions of 1.1 kb to 10 kb [52,59]. The range was similarly broad in those with KSS who developed adrenal insufficiency (3 kb to 9 kb) [58]. The most common deletion in KSS is ~5 kb [58,69]. Again, only one patient with adrenal insufficiency had a deletion around this size (4.9 kb).

As there was a wide range in deletion sizes in the patients with adrenal insufficiency, it is unlikely that the deletion length or mtDNA genes affected particularly correlate with adrenal dysfunction. This aligns with previous findings that indicate neither the size nor the location of mtDNA deletions that determines the clinical course, rather, it is the distribution of mutant mitochondria between different tissues [70]. However, this is also contested within the literature and the exact pathogenesis of KSS remains unsolved [55]. Interestingly, although ocular involvement is typical for KSS (see Appendix B), most people with KSS and adrenal insufficiency did not have ophthalmic problems [52]. Similarly cardiac pathologies are also common in KSS, but again, were only described in two patients with adrenal insufficiency [52,58]. As with PS, this non-classical presentation may result from non-classical mtDNA deletions.

Atypical phenotypes in people with mitochondrial deletion syndromes and adrenal failure present an additional diagnostic challenge. Adrenal insufficiency may easily be overlooked in people presenting with generalised systemic complaints. Additionally, this review demonstrates that adrenal dysfunction is infrequently investigated or described in people with mitochondropathies [55]. Thus, there may be some element of under-detection and underreporting, which contributes to the scarcity of case reports in the literature (see Table 1). Alternatively, the adrenal insufficiency may be due to undetected mutations in genes known to cause primary adrenal failure where whole exome/genome analysis has not been undertaken.

### 9.2. Non-Deletion Mutations

Adrenal insufficiency also occurred in patients with mitochondropathies due to mtDNA point mutations [4,53]. There are two reported cases of PAI alongside MELAS, which is characterised by myopathy, encephalopathy, lactic acidosis, and stroke-like episodes [4,47,71]. Adrenal dysfunction was not the presenting complaint in either of these cases, and instead, developed later in the disease progression [47,66,71].

Over 80% of MELAS cases arise from a single mtDNA missense point mutation (m.3243A>G) [47]. This impairs the expression of mitochondrial tRNA and consequently reduces vital mitochondrial protein formation and cellular oxidative phosphorylation [47]. Interestingly, this m.3243A>G variant was not found in either of the cases with adrenal insufficiency, supporting the notion that the AI is of a different origin [4,47,71].

Outside of MELAS, other monogenetic mutations affecting various mitochondrial components have been linked to PAI. Calderwood et al. reported a novel *GFER* mutation that caused an absolute loss of function of ETC complex I [4]. Similarly, Endres et al. uncovered a *MT-ND4* variant that led to ETC complex 1 subunit malformation [47]. Dursun et al. uncovered a novel biallelic QRSL1 mutation that decreased the activity of ETC Complexes I, II, IV, and V [65]. The increased oxidative stress and mitochondrial damage may have resulted in their patient’s severe PAI. However, there was no further molecular investigation of the adrenal dysfunction, and so it is currently unclear how, or if, these mtDNA variants contribute to the deranged cortisol biosynthesis. Similarly, there were nine other cases of monogenetic mtDNA mutations with adrenal insufficiency where the underlying pathogenesis of cortisol deficiency remains elusive [48,67,68]. In four of these cases, the variant impaired mtDNA expression, and consequently, the synthesis of key mitochondrial proteins [48,67,68]. This will have affected multiple mitochondrial biochemical pathways, potentially including the ROS detoxification systems. Thus, ROS accumulation and oxidative stress may underly adrenal dysfunction in these cases.

It is also possible that there were more mutated mitochondria in the adrenals than in other tissues. However, the relationship between mitochondrial heteroplasmy and phenotypic presentation is debated [55]. Boles et al. reported a case of KSS with a clinical picture dominated by adrenal insufficiency [55]. However, the penetrance of mutated mitochondria in the adrenals was only 65% compared to 95% and 83% in the liver and pancreas, respectively, which both had near-normal function [55].

It is evident from the paucity of case reports in the literature that mitochondrial dysfunction (particularly ROS accumulation) is likely not to be solely responsible for adrenal insufficiency in mitochondropathies.

## 10. Future Research

As demonstrated, mitochondrial disorders are highly heterogeneous and are likely underreported. More research is needed into the pathogenesis of adrenal disease in these individuals. Data mining of their genomes may reveal the presence of previously undiscovered genetic variants in genes known to cause PAI, but, if not, may reveal the relative role of mitochondrial defects in adrenal insufficiency. These discoveries could have huge diagnostic and prognostic benefits given the potentially lethal consequences of untreated adrenal insufficiency.

## Figures and Tables

**Table 1:** Summary of all reported cases of adrenal insufficiency in mitochondrial diseases.

Author	Year	Gene Affected/ Mutation	Resultant Pathology	Diagnosis	Gender	Age of PAI	Other Clinical Symptoms	Impaired MC	Impaired GC	PAI?	Other Endocrine Dysfunction?
Ribes	1993	1.5 kb mtDNA deletion	Complex 4 deficiency	Pearson	Male		Metabolic derangement and acidosis, bilateral corneal opacities, anaemia	Y	Y	Unknown	Unknown
Nicolino	1997	7.436 kb mtDNA deletion	Complex 1 deficiency, impaired mtDNA translation	Not stated	Female	4	Short stature, cognitive decline, sensorineural hearing loss, ophthalmic involvement, impaired glucose tolerance	Y	Y	Y	Y
Artuch	1998	6.7 kb mtDNA deletion	Loss of 10–45% mtDNA	KSS	Male	16	Bilateral degenerative retinopathy, developmental delay, complete heart block, T2DM, gynecomastia, obesity, hyperpigmentation	Unknown	Y	Autoimmune	Y
Artuch	1998	6.7 kb mtDNA deletion	Loss of 64–66% mtDNA	KSS	Male	7	hyperpigmentation, growth retardation, ptosis, hypoparathyroidism	Unknown	Y	Y	Y
Boles	1998	4.9 kb mtDNA deletion	65% mutated adrenal mtDNA, trace scar tissue in adrenals	KSS	Male	5	Growth retardation, RTA, developmental delay, ophthalmoplegia, hyperpigmentation, lactic acidosis,	Unknown	Y	Y	N
Bruno	1998	6.9 kb mtDNA deletion	Complex 1 & 4 deficiency	Not stated	Female	4	Developmental delay, hyperpigmentation, growth retardation, hypotonia, lactic acidosis, hypoparathyroidism	N	Y	Y	Y
Sanaker	2007	3–4 kb mtDNA deletion	Impaired Oxidative Phosphorylation	KSS	Female	32	Ophthalmoplegia, short stature, RBBB, myopathy, hypothyroidism, Type 2 respiratory failure	Y	Y	Autoimmune	Y
Sugiana	2008	*NDUFAF5* gene—c.719C>T (C20orf)	Impaired complex 1 assembly	Not stated	Male	7 d	Lactic acidosis, congenital abnormalities, hypotension	Unknown	Y	Y	N
Hopkins	2010	*POLG1* gene—c.1550G>T (p.G517V)	N/A	Not stated	Female	10	Type 1 Diabetes, hypothyroidism, seizures, chorea, psychiatric involvement, hypercalcaemia	Unknown	Unknown	Autoimmune	Y
Hopkins	2010	*POLG1* gene—c.1550G>T (p.G517V)	N/A	Not stated	Female	11	Type 1 diabetes, hypothyroidism, bilateral basal ganglia pathologies, psychiatric involvement, hypercalcaemia	Unknown	Unknown	Autoimmune	Y
Duran	2011	7.3 kb mtDNA deletion	N/A	Not stated	Male	3	Short stature, hyperpigmentation, chronic pancreatitis, congenital glaucoma	N	Y	Autoimmune	Y
Tzoufi	2012	9 kb mtDNA deletion	N/A	KSS	Male	5	Lactic acidosis, myopathy, hyperpigmentation, hypoparathyroidism, Fanconi syndrome	Y	Y	Y	Y
Williams	2012	5.1 kb mtDNA deletion	N/A	Pearson	Male	4	Anaemia, febrile seizures, hyperpigmentation, hypertonia, lactic acidosis, leucopoenia, thrombocytopaenia	Y	Y	Y	N
Afroze	2014	m.8344A>G	N/A	MELAS	Male	5	Febrile seizures, hyperpigmentation, hypertonia, lactic acidosis	N	Y	Y	N
Menes	2015	*MRPS7* gene—c.550A>G (p.Met184Val)	Impaired mtDNA translation	Not stated	Female	16	Sensorineural deafness, lactic acidosis, primary hypogonadism	Unknown	Y	Y	Y
North, Calderwood	1996, 2015	*GFER* gene—c.581 G>A (R194 H), c.373 C>T	No complex 1 activity, Impaired 2,3,4 activity	Mitochondrial encephalo-myopathy	Female	7 m	Lactic acidosis, growth retardation, bilateral cataracts, hyperpigmentation, hepatomegaly	N	Y	Y	N
O’Grady	2015	Unknown	Complex 4 deficiency	Not Stated	Male	13	Sideroblastic anaemia, neurological dysfunction, renal tubulopathy, faltering growth, Fanconi syndrome, T1DM	Y	Y	Y	Y
Kohda	2016	*QRSL1* gene—c.398G>T (p.G133V)	I, II, III, and IV complex deficiencies	lethal infantile mitochondrial disease (LIMD)	Female	<1 m	Hypertrophic cardiomyopathy, hearing loss	Unknown	Y	Unknown	N
Farruggia	2016	Not described	Unknown	Pearson	Unknown	2.9	Hepatomegaly, growth impairment, ventricular wall thickness, neurological involvement, severe infections, hyperlactatemia	Y	Y	Unknown	N
Farruggia	2016	Not described	Unknown	Pearson	Unknown		Hepatomegaly, splenomegaly, ventricular wall thickness, severe infections, hyperlactatemia	Y	Y	Unknown	N
Vona	2018	*IARS2* gene—c.2725C>T (p.Pro909Ser)	mt-tRNA malformation	CAGSSS	Male	20.6	Congenital cataracts, short stature, GH deficiency, sensorineural hearing loss, peripheral neuropathy, Type II achalasia	N	Y	Y	Y
Endres	2019	*MT-ND4* gene—m.12015T>C (p.Leu419Pro)	Complex 1 subunit malformation	MELAS	Female	25	Dysexecutive syndrome, muscular fatigue, Hashimoto’s thyroiditis	Y	y	Autoimmune	Y
Son Soo	2022	2.3 kb mtDNA deletion	Unknown	Pearson	Female	6	Seizures, ketotic hypoglycaemia, failure to thrive, pancreatic insufficiency, macrocytic anaemia	Unknown	Y	Unknown	Y
Dursun	2022	c.300T>A; Y100 * and c.610G>A; G204R	QRSL1	COXPD40	Male	6	Developmental delay sensorineural deafness, cardiomyopathy, renal impairment, lactic acidosis, hypertension	N	Y	Y	N

Y = yes, N = no, RTA = renal tubular acidosis, RBBB = right bundle branch block, T2DM = Type 2 diabetes mellitus, COXPD40 = ‘combined oxidative phosphorylation deficiency-40’, * = termination codon.

## Data Availability

Not applicable.

## Appendix A. Familial Glucocorticoid Deficiency

### Appendix A.1. Familial Glucocorticoid Deficiency (FGD)

Given the central role of mitochondria in steroidogenesis, it is unsurprising that mutations in mitochondrial regulatory genes lead to diseases with cortisol deficiency such as FGD [2,8,11,20,23,30].

### Appendix A.2. Incidence and Presentation

FGD is a rare autosomal recessive heterogeneous disease. FGD is characterised by isolated GC deficiency due to ACTH resistance [8,11,15,41]. Serum cortisol levels are often undetectable with very high ACTH, but normal mineralocorticoid concentrations and electrolytes [8,11,12,41].

Symptom severity varies, with individuals presenting throughout early infancy and childhood with general systemic symptoms such as pallor and failure to thrive [8,15,41]. Classical features result from cortisol deficiency including acute adrenal failure and hypoglycaemia [8].

Non-specific symptoms may obscure diagnosis, with potentially lethal consequences [41]. Chronic hypoglycaemia, resultant seizures, and repeated infections throughout childhood have serious neurological sequelae, ranging from learning difficulties to premature death [3,41]. Additionally, treatment for comorbid conditions (such as inhaled corticosteroids for asthma) may also delay diagnosis [41]. A high index of suspicion of FGD is recommended for any child presenting with non-specific symptoms and adrenal crisis [3,16,41,72].

### Appendix A.3. Diagnosis and Management

ACTH resistance causes a low serum morning cortisol (<80 nmol/L) with excessively high ACTH (more than double upper reference limit) [3,41,72]. Diagnosis is confirmed biochemically through a corticotropin stimulation test demonstrating persistent low cortisol [3,4]. Lifelong hydrocortisone replacement therapy is the mainstay of treatment, and carries a good prognosis, often resolving most symptoms (3,4,12,41). Genetic analysis is also recommended for all people with FGD to guide genetic counselling and optimise future management [3,16,41].

## Appendix B. Kearns–Sayre Syndrome and Pearson Syndrome

Pearson syndrome is a sporadic genetic disease that typically presents in infancy or early childhood [59]. Prognosis is poor, with few surviving into early adulthood. Those that do often subsequently develop other diseases such as KSS [73]. Classical symptoms are failure to thrive, growth retardation, and developmental delay [52,59]. Liver derangement, lactic acidosis, and renal dysfunction are also common [52,59].

Similarly, Kearns–Sayre syndrome (KSS) presents in young people (under 20) and is often due to de novo mtDNA deletions [58]. Characteristic features are progressive opthalmoplegia with pigmented retinopathy and encephalopathies, which often occur alongside progressive wider organ system failure or dysfunction [58].
